# Intravesical human papillomavirus (IHPV) infection–endoscopic resection

**DOI:** 10.1590/S1677-5538.IBJU.2015.0016

**Published:** 2016

**Authors:** Silvio Henrique Maia Almeida, Marco Aurélio Cruciol Rodrigues, Daniele Mathiel, Susana Fonseca Alves

**Affiliations:** 1 Departamento de Cirurgia - Universidade Estadual de Londrina, Londrina, PR, Brasil

## INTRODUCTION

Although HPV infections are common, intravesical HPV is a rare condition, with only 20 reported cases (1).

Objectives: The aim of this video is to present a case treated with transurethral resection (TR).

## RESULTS

A 38-year-old woman, with a history of urinary tract infections. She denied other diseases and complained of painful urination. The genital examination, cervical cytology and colposcopy were normal. Urinalysis showed leukocytosis, but a negative culture. The ultrasound was normal. A cystoscopy was performed, showing polypoid growth on the bladder mucosa followed of the TR. Histopathologic analysis (HA) confirmed IHPV (in situ hybridization positive for 6, 18, 31 subtypes), associated a Bladder Squamous Metaplasia (BSM). The patient recurrence-free was for 5 years when a cystoscopy demonstrated BSM, which was resected. The patient had not any other sites (urethral, genital or anal) of recurrence during the period of follow-up.

## DISCUSSION

The condyloma, caused by types 6 and 11, is more frequent in the genital mucosa and anal area (1, 2). IHPV presents with a polypoid pattern, often related to warts in the urethra. The clinical presentation includes hematuria, dysuria, fever and pelvic pain (1, 2). HA evaluation shows hyperproliferation of metaplasic squamous cells, the transition to the normal epithelium is sudden, with no submucosal invasion. This pattern excludes squamous cell carcinoma (SCC). A standard treatment was not established. TR has also been successfully used (1), and radical cystectomy was indicated when the lesion was associated with SCC (3). Since the cytotoxic immune responses against malignant and virally transformed cells appear to be similar, local application of BCG might also be effective in the adjuvant treatment of recurrent condylomata (4).

## CONCLUSIONS

TR may be a safe option for definitive IHPV treatment. However, the patients must be followed as in cases of the superficial carcinoma of transitional epithelium.

## ARTICLE INFO

**Available at: ARTICLE INFO Available at:** **http://http://www.intbrazjurol.com.br/video-section/almeida_395_396/**

**Int Braz J Urol. 2016; 42 (Video #3): 395-6**

## References

[1] Massé S, Tosi-Kruse A, Carmel M, Elhilali M (1981). Condyloma acuminatum of bladder. Urology.

[2] Barber NJ, Kane TP, Conroy B, Britton JP (2003). Condyloma acuminatum-like lesion of the urinary bladder progressing to invasive spindle cell carcinoma. Scand J Urol Nephrol.

[3] White R, Donnellan S, Leong T (2011). Complete resolution of urinary bladder condyloma acuminatum following definitive chemoradiotherapy for anal cancer. BJU Int.

[4] Böhle A, Doehn C, Kausch I, Jocham D (1998). Treatment of recurrent penile condylomata acuminata with external application and intraurethral instillation of bacillus Calmette-Guerin. J Urol.

