# Supplementary material for: Intravesical human papillomavirus (IHPV) infection–endoscopic resection
Source: Int Braz J Urol. 2016 Mar-Apr;42(2):395–6. doi: 10.1590/S1677-5538.IBJU.2015.0016 (PMC4871405; doi:10.1590/S1677-5538.IBJU.2015.0016)
Supplement: Related texts

# Letter: EDITORIAL COMMENT

**Authors:** Pranav Sharma

**Affiliations:** Department of Genitourinary Oncology, Moffitt Cancer Center, Tampa, FL, USA, Email: pranav.sharma@moffitt.org

This interesting video by Almieda and colleagues highlights a rare occurrence of HPV infection within the bladder mucosa diagnosed after transurethral resection. It further characterizes the diffuse nature of HPV-associated manifestations, which can cause cancer at multiple anatomic sites in men and women, including cervical, oropharyngeal, anal, vuluvar, vaginal, and penile malignancies. In this particularly case, the patient remained recurrence-free for 5 years, which reinforces the heterogeneity of HPV-associated disease and its malignant potential based on the anatomic site of infection (1). We applaud the authors for their novel work, which adds to the body of literature regarding potential sites of HPV-related disease and its differing natural history based on the site of infection.

## References

[1] Giuliano AR, Nyitray AG, Kreimer AR, Pierce Campbell CM, Goodman MT, Sudenga SL (2015). EUROGIN 2014 roadmap: differences in human papillomavirus infection natural history, transmission and human papillomavirus-related cancer incidence by gender and anatomic site of infection. Int J Cancer.
